# Treatment Landscape of Metabolic-Dysfunction-Associated Steatotic Liver Disease

**DOI:** 10.3390/jcm14176060

**Published:** 2025-08-27

**Authors:** Pranav Patel

**Affiliations:** Prisma Health Greenville Memorial Hospital, Greenville, SC 29605, USA; pranav.patel@prismahealth.org

**Keywords:** MASLD, fatty liver disease, treatment of MASLD, novel therapeutics in MASLD, GLP-1, FGF-analogs, resmetirom, lanifibranor, SGLT-2s, novel therapeutics

## Abstract

**Background/Objectives**: The incidence of metabolic-dysfunction-associated steatotic liver disease (MASLD) is on the rise worldwide. The purpose of this paper is to review the current and emerging trends in the management and treatment of this condition. **Methods**: A comprehensive literature review was conducted using PubMed and GoogleScholar, focusing on articles published within the last ten years. **Results**: As the incidence of MASLD rises worldwide, it is becoming ever more important to call attention to disease prevention and progression. Although weight loss, diet, and exercise play a major role, certain therapies including GLP-1 receptor agonists, resmetirom, lanifibranor, and FGF-3 analogs are showing promise when treating patients with MASLD. As more drugs become available, it will be important to note how these medications change the global outlook of this disease. **Conclusions**: Overall, the treatment landscape of MASLD is rapidly changing. Several phase 3 trials have revealed promising data when it comes to improving liver fibrosis and histology. This shift in treatment will provide new hope for patients and clinicians when treating this challenging disease.

## 1. Introduction

Metabolic dysfunction-associated steatotic liver disease (MASLD) is one of the most common forms of chronic liver diseases worldwide. As the rates of obesity have risen over the past few decades, the risk of developing MASLD continues to increase. Previously, steatotic liver disease was defined as non-alcoholic fatty liver disease (NAFLD) but with recent advances in research showing a correlation between metabolic syndrome and NAFLD, the nomenclature has been updated to reflect this association [1].

As the incidence of obesity, metabolic syndrome, and type 2 diabetes mellitus have increased in the United States, so has the incidence of MASLD. In 2020, it was estimated that 33.7% of the United States adult population had MASLD, and this figure is projected to increase to 36.8% by 2030 [2]. The increasing prevalence of the disease will lead to higher healthcare utilization, poor patient outcomes, and an overall decrease in quality of life.

In addition to metabolic risk factors, emerging evidence has identified gut dysbiosis as another key driver of MASLD pathogenesis. Alterations in the gut microbiome, characterized by reductions in beneficial short-chain fatty acid-producing bacteria and overrepresentation of pro-inflammatory species, compromise intestinal barrier function and increase permeability. This promotes the hepatic influx of microbial products which trigger hepatic inflammation, insulin resistance, and fibrogenesis—central processes in MASLD progression [3].

Thus, it is crucial to highlight the importance of screening and educating patients at risk of developing MASLD. In order to improve patient outcomes, a multi-disciplinary approach will be key. The purpose of this review is to highlight the current treatment landscape of metabolic-associated steatotic fatty liver disease (MASLD) and discuss areas of future research.

## 2. Materials and Methods

A comprehensive literature search was conducted on 6 May 2025, using PubMed (https://www.ncbi.nlm.nih.gov/) and Google Scholar databases (Greenville, SC, USA). Search terms included: “new treatments for MASLD,” “Clinical trials for fatty liver disease,” “treatment outcomes,” “current clinical trials for MASLD treatment”, and “epidemiology of MASLD.” The search was limited to articles published in the previous 10 years (2015–2025), written in English, and involving human subjects. ChatGPT 2.0 was utilized to make Table 1.

A total of 36 articles were initially retrieved. After title and abstract screening, 25 full-text articles were assessed for eligibility. Ultimately, 15 studies were included in this review, based on the following criteria. Studies that focused on epidemiology, management, current clinical trials, and treatments were evaluated. Studies providing updated clinical guidelines or recent public health data were also included. Non-English language publications and publications older than 10 years were excluded.

## 3. Results

### 3.1. Background

The incidence of metabolic dysfunction-associated steatotic liver disease (MASLD) is rapidly increasing [4]. Currently, MASLD is increasing in parallel to the increasing rates of obesity and type 2 diabetes (Type 2 DM) and it is currently the most common form of chronic liver disease amongst adults [4]. According to recent data, the global prevalence of fatty liver disease has increased from 25.3% in 1990–2006 to 38% in 2016–2019 [4]. As the disease burden rises, it will become even more important to incorporate preventative strategies, in addition to continually evaluating patients for advanced therapies.

MASLD is an overarching term used to characterize a condition where there is evidence of hepatic steatosis (>5% hepatic steatosis) on imaging or histology (macro-vesicular steatosis) with the absence of secondary readily identifiable causes of steatosis [4,5]. Those secondary causes include the absence of significant alcohol consumption, starvation, medications, or hereditary disorders that may contribute to the development of liver disorders. The definition of significant alcohol use includes <20 g/day for women and <30 g/day for men [6]. Steatotic (fatty) liver disease is a comprehensive term that may be further classified as MASLD (metabolic dysfunction-associated steatotic liver disease), which includes a fatty liver with at least one metabolic risk factor such as dyslipidemia or obesity [1,5,6]. This category was formerly known as NAFLD (non-alcoholic fatty liver disease). MASLD with MASH (metabolic dysfunction-associated steatohepatitis) is defined by histologic evidence of inflammation, hepatocellular injury such as ballooning of hepatocytes with or without fibrosis, which was previously known as non-alcoholic steatohepatitis (NASH) [1,6,7]. It is important to note the differences between the two, as the nomenclatures for each disease have been updated.

Patients at risk of developing MASLD go through several phases of progression. The progression of disease starts with simple steatosis, steatohepatitis, fibrosis, and ultimately, cirrhosis [4]. Although the disease course is benign, the more advanced forms of disease can have many long-term implications in affected patients.

It is key to note that several factors increase the risk for developing MASLD. Since the correlation between cardiac risk factors and MASLD was established, people with type 2 DM, hyperlipidemia, central obesity and hypertension are at the highest risk for developing progressive liver disease [6]. In addition, having several of these metabolic abnormalities concurrently confers an even greater risk for histological progression of MASLD [6].

**Figure 1:**
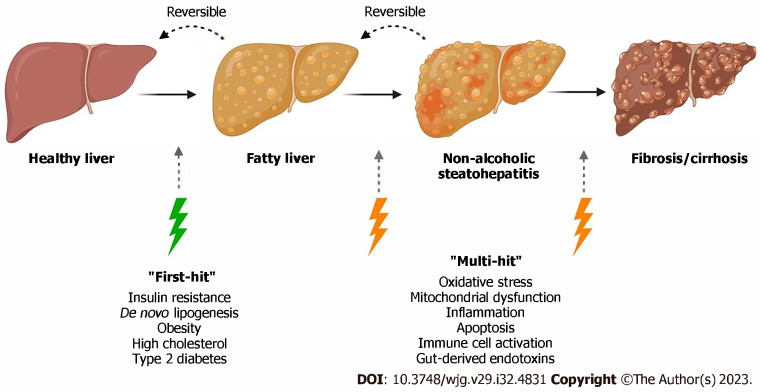
**Shows the progression of normal liver to liver fibrosis.** In the “multiple-hit” theory of progression, the first cause or “first hit” in NAFLD is insulin resistance, obesity, type 2 diabetes, and metabolic syndrome. As the first hit occurs, free fatty acids are stored in the liver as triglycerides, resulting in simple steatosis. Disease progresses when multiple factors, or “multi-hits”, such as oxidative stress, inflammatory mediators, apoptosis, and mitochondrial dysfunction cause liver damage.

As the burden of fatty liver disease rises worldwide, it is crucial for clinicians to recognize and implement preventative and lifestyle measures to stop progression. For most patients, the cornerstones of treatment include a healthy diet, minimizing alcohol intake, and exercise [6]. Even if weight loss is not needed, improved diet and increased exercise can promote cardiovascular health in addition to improved control of metabolic co-morbidities [6]. There are specific drugs that have shown a benefit in treating patients with MASLD. First, this paper discusses drugs that were originally targeted for Type 2 DM such as glucagon-like peptide 1 receptor agonist, pioglitazone, and sodium-glucose transport 2 inhibitors, which are now being considered for treating fatty liver disease. We also discuss specific drugs such as resmetiron, fibroblast growth-factor 21 analogs, and lanifibranor, as these are currently undergoing phase 3 clinical trials and showing great promise treating patients suffering from MASLD. Refer to Figure 1 for a brief overview of the pathophysiology leading up to MASH and eventually liver cirrhosis.

Adapted from Petagine, et al. Reproduced under the Creative Commons Attribution License [7].

### 3.2. Benefits of Lifestyle Modifications

The majority of patients who are initially diagnosed with MASLD are treated with conservative management alone. As noted, a strong association between cardiac risk factors and the development of MASLD has been established. Thus, a set of general guidelines should be followed when recommending initial treatment. Management should start with weight loss as the primary therapy for MASLD [8]. It is recommended that patients who are overweight or have obesity lose about 5–7% of body weight which is about 1–2 lbs. per week. Patients with suspected or biopsy-proven MASH (metabolic associated steatohepatitis), the weight loss goal is higher to 7–10% of body weight [6]. It has been shown that weight loss > 7% improves histological disease activity [9]. Thus, it remains crucial for patients to make lifestyle changes to achieve this goal.

An important characteristic to achieve these targeted weight loss goals is incorporating regular exercise. The American College of Sports Medicine (ACSM) recommends at least 150 min/weekly of moderate or 75 min/weekly of vigorous-intensity physical activity for all patients with MASLD [10]. In return, this not only assists with weight loss but also increases insulin sensitivity and decreases free fatty acids and de novo lipogenesis [10], all of which play a role in the pathophysiology of developing MASLD.

For patients who do not meet their weight loss goals after 6 months, other options such as bariatric surgery can be discussed. In a recent observational study, a cohort of 431 participants were biopsy screened for histological MASLD [11]. Out of the 288 that qualified for the study, 156 participants underwent Rou-en-Y gastric bypass or sleeve gastrectomy, while the others were assigned to lifestyle modifications only [9]. Compared with lifestyle modifications alone, patients who underwent a bariatric procedure had a 3.6 times higher likelihood of MASH resolution [11]. Additionally, patients who underwent bariatric surgery also had a lower risk of developing new-onset heart failure, cerebrovascular events, and coronary artery interventions [12].

### 3.3. Diabetic Drugs Targeting MASH

Alongside weight loss and exercise, medical therapies including anti-diabetic drugs are rapidly gaining ground. These medications are becoming popular as they help control certain metabolic factors such as diabetes and lipid metabolism [13]. These factors play a large role in developing metabolic syndrome and are directly implicated in MASLD development.

#### 3.3.1. Glucagon-like Peptide-1 Receptor Agonists (GLP-1 RA)

Glucagon-like peptide-1 receptor agonists (GLP-1 RA’s) have become increasingly popular as a treatment for diabetes and weight loss. GLP-1 RA is an incretin hormone that stimulates the release of insulin from pancreatic beta-cells in response to carbohydrates that are absorbed from the gut [14]. It also controls glucose homeostasis by delaying gastric emptying which in return helps control post-prandial hyperglycemia. Furthermore, GLP-1 RA’s affect specific portions of the hypothalamus where hunger is controlled and as a result, patients benefit from weight loss due to reduced caloric intake [14]. By exerting such effects, patients can control their metabolic risk factors and reduce their chances of developing MASLD.

There has been ongoing research on the positive effects of GLP-1s and steatohepatitis. Semaglutide is part of an ongoing phase 3 clinical (ESSENCE) trial where its benefits on reducing biopsy proven steatohepatitis and liver fibrosis stage 2 or 3 is being studied [15]. The primary end point of the study is the resolution of steatohepatitis and reduction of liver fibrosis without worsening steatohepatitis. Resolution of steatohepatitis was seen in 62.9% of the 534 patients in the semaglutide group versus 34.4% of the 266 patients in the placebo group [15]. In addition, reduction in liver fibrosis without worsening of steatohepatitis was noted in 36.8% of patients in the semaglutide group and in 22.4% of those in the placebo group. In patients who took semaglutide, the reduction of steatohepatitis and moderate to advanced fibrosis proved to be beneficial when compared with placebo [15].

Clinical trials that have been completed on other GLP-1 RAs have also demonstrated significant clinical benefit. Tirzepatide, which is a dual GLP-1 and glucose-independent insulinotropic polypeptide (GIP), has shown even greater efficacy in weight loss than semaglutide. The SURMOUNT—5 study included a direct head-to-head comparison of semaglutide vs. tirzepatide. In that study, the primary end point was to measure the greatest percent change in weight from week 1 to week 72 [16]. The study only included individuals with obesity but without diabetes, enrolling 751 participants who underwent randomization. The end point of the study revealed that among participants who took both medications, tirzepatide was superior to semaglutide when it came to reduction in body weight and waist circumference [16]. Although the study did not reveal direct effects on patients with MASLD, its results suggest favorable outcomes for patients with at least one metabolic risk factor leading to fatty liver disease.

The benefits of GLP-1/glucagon receptor co-agonists have also undergone extensive research. Cotadutide is a dual GLP-1 and glucagon receptor agonist that has shown benefit in patients suffering from MASLD. In a phase 2b study (NCT03235050), Cotadutide was assessed for HbA1c reduction and improved body weight by week 14 [17]. Liver biomarkers and liver fibrosis scores were also assessed. Participants in a randomized double-blind study were assigned to either a placebo group or cotadutide group or an open label liraglutide group [17]. It was shown that cotadutide significantly decreased A1c levels and body weight at weeks 14 and 54. Improvements in AST and ALT levels and fibrosis—4 index were observed in the cotadutide 300 ug vs. placebo group, but not with liraglutide. It is important to note that weight loss, reduction in HbA1c and liver biomarkers were observed in a dose-dependent fashion with cotadutide [17].

Survodutide is a glucagon/GLP-1 receptor dual agonist that is also showing great promise. In 2024, the US FDA gave the drug a breakthrough designation status to study the benefits of this therapy for patients living with MASH (metabolic associated steato-hepatitis) and fibrosis and MASH with compensated liver cirrhosis [18]. In its phase 2 study, survodutide proved to be superior when compared to placebo with respect to improvement in MASH, without worsening of fibrosis when compared to placebo, warranting further investigation in phase 3 trials [19]. The phase III clinical trial was launched into two separate studies: the LIVERAGE trial designated for patients with MASH with moderate to advanced fibrosis and the LIVERAGE—Cirrhosis trial for those with MASH with compensated liver cirrhosis [18].

Efinopegdutide and pemvidutide represent another class of combined glucagon dual receptor agonists/GLP-1 medications that has been studied recently. In a phase II randomized trial, efinopegdutide 10 mg was associated with a statistically significant decrease in liver fat content in patients with MASH compared with semaglutide 1 mg once weekly for 24 weeks [20]. Additionally, greater reductions in other metabolic risk factors such as HDL, LDL, TGL levels, and body weight were noted in the efinopegdutide group compared with semaglutide [19]. Additionally, pemvidutide (1.2 mg, 1.8 mg, 2.4 mg once weekly) was compared to placebo; the 1.2 mg and 1.8 mg groups demonstrated significant reduction in liver fat content [19].

Although GLP-1 medications are starting to become the forefront of treating fatty liver disease, it is important to note the drug class’s side effects. Due to its mechanism of action, GLP-1s exert many effects on the gastrointestinal tract that can lead to poorer outcomes or complete intolerance by patients. The most common side effects include abdominal pain, constipation, diarrhea, nausea, and vomiting [21]. It is important to note that the risk of pancreatitis and gastroparesis also remains in patients taking GLP-1s, but the incidence remains low. In a recent retrospective review, GI side effects were more common in women than in men and more prevalent in patients with chronic kidney disease (CKD) and heart failure [21]. As a result, prescribing clinicians will have to constantly educate patients to ensure proper compliance and safety to promote long term adherence.

In summary, GLP-1 receptor agonists and their dual-agonist counterparts represent a promising class of therapies for patients with MASLD and MASH. By targeting multiple metabolic pathways ranging from glycemic control and appetite suppression to direct hepatic effects, these agents have demonstrated significant benefits in reducing liver fat, improving liver enzyme profiles, and even reversing steatohepatitis and fibrosis in clinical trials. As ongoing phase 3 studies continue to evaluate long-term efficacy and safety, these therapies may soon become integral components of a multidisciplinary approach to managing MASLD.

#### 3.3.2. Pioglitazone

Pioglitazone is a thiazolidinedione which increases insulin sensitivity and lipid metabolism by acting on peroxisome proliferator-activated receptor gamma (PPARγ) receptors. It reduces insulin resistance by improving lipid storage/redistribution and glucose utilization [21]. Pioglitazone was one of the first anti-diabetic drugs to show promise when it came to treating patients with MASLD [22]. In a randomized clinical trial (RCT) involving 55 people with pre-diabetes/type 2 DM and biopsy-proven MASH, it showed a significant difference when compared with placebo in reducing liver fat content and improvement in histologic findings [23,24]. In a meta-analysis of eight RCTs involving 516 people with biopsy proven MASH, pioglitazone was noted to have improved advanced fibrosis of any stage and MASH resolution in patients. Despite showing great benefits, pioglitazone has been implicated with adverse events such as heart failure, weight gain and increased fracture risk which has made the drug less favorable [22]. As a result, pioglitazone is not routinely recommended for use in patients with MASLD.

#### 3.3.3. Sodium-Glucose Co-Transporter-2 Inhibitors (SGLT-2 Inhibitors)

Sodium-glucose co-transporter- 2 inhibitors (SGLT-2 inhibitors) have shown promise in treating patients with MASLD. The drug has already been proven to reduce the risk of chronic kidney disease (CKD) progression, reduce heart failure exacerbations and improve diabetic outcomes. These agents work by inhibiting the reabsorption of glucose in the proximal renal tubules to facilitate urinary glucose excretion [25]. This glycemic control, combined with weight loss has made this drug class an important area of study [25]. In addition, SGLT-2 inhibitors can decrease plasma triglyceride levels and increase high-density lipoprotein which can improve dyslipidemia and be helpful in alleviating hepatic steatosis.

A recent meta-analysis reviewed 18 eligible RCTs involving 1330 participants and based on those results, SGLT-2 inhibitors were shown to slightly improve hepatic steatosis and fibrosis when compared to controls with low to moderate certainty of evidence [26]. This study included: empagliflozin, dapagliflozin, tofogliflozin, luseogliflozin, licogliflozin and ipragliflozin. Despite showing promise, SGLT-2 inhibitors need more randomized controlled trials and a larger sample size before they can be considered as a standard of treatment for MASLD patients.

#### 3.3.4. Vitamin E

Vitamin E, a potent anti-oxidant, has also been considered as a potential therapeutic option for treating non-diabetic patients with MASLD. It manages to prevent liver injury by reducing oxidative stress. In the setting of metabolic syndrome, the increased delivery of fatty acids to the liver results in amplified oxidative stress through fatty acid oxidation and oxidative phosphorylation [27]. This produces an environment high in reactive oxygen species which can cause hepatocyte injury and hepatic damage. Vitamin E can reduce oxidative stress via various pathways, which directly aids in improving hepatic steatosis, lobular inflammation and hepatocyte ballooning [27].

In a recent double-blind, randomized trial by Song et. al, Vitamin E was compared to placebo. The study included 124 non-diabetic patients with biopsy proven MASH. When compared with placebo, the patients who took Vitamin E 300 mg had significant improvement in steatosis, lobular inflammation and liver fibrosis [28]. In addition, a systematic review of 11 studies concluded that patients who took Vitamin E 800 IU daily noticed improvement in liver enzymes (ALT and AST), hepatic steatosis, and inflammation [29]. Although it showed promising results in those areas, it did not have significant impact on liver fibrosis. Despite initial success, the studies that have been conducted for Vitamin E have been limited in size which calls for future research prior to being considered as routine therapy for MASLD.

## 4. Novel Therapeutics for MASLD

### 4.1. Resmetirom

The therapeutic landscape for MASLD continues to evolve, with resmetirom emerging as one of the newest FDA-approved agents for patients with moderate to advanced fibrosis. Approved in 2024, resmetiron exerts its effects by targeting THR-β (thyroid hormone receptor—beta) selective receptor on the liver [30]. THR-β is responsible for regulating metabolic pathways in the liver and it is frequently impaired in MASLD patients [30]. As a result, this affects lipid metabolism, fatty acid oxidation and energy production which can potentially worsen MASLD and liver fibrosis. The lipotoxicity that occurs in fatty liver disease induces intrahepatic hypothyroidism resulting in reduced conversion of pro-hormone T4 to active T3. Instead, there is an increased conversion of T4 to the inactive metabolite reverse T3 (rT3). By targeting the thyroid hormone receptor, Resmetirom proved to be efficacious in MASLD resolution and in improving fibrosis by at least one stage without any significant worsening in MASLD activity score [30].

This pivotal data was revealed from the MAESTRO-NASH trial where 1759 participants underwent a double blind, placebo-controlled trial. After 52 weeks of treatment, both 100 mg and 80 mg of resmetirom demonstrated significant improvement in the treatment arm when compared with placebo. The primary end-points of the study were MASLD resolution (including a reduction in disease activity score ≥ 2 points) with no worsening fibrosis and an improvement in fibrosis by at least one stage without evidence of worsening in the disease activity score [30]. Despite its promising efficacy, the cost and accessibility of Resmetirom may limit widespread adoption. While the manufacturer offers financial assistance programs, broader use is likely to depend on commercial insurance coverage. 

### 4.2. Lanifibranor

Lanifibranor has emerged as one of the most promising investigational therapies for managing metabolic associated steatohepatitis (MASH). Although not yet FDA-approved, the drug gained early momentum in 2020 when it received breakthrough therapy designation from the U.S. Food and Drug Administration [31]. This designation is reserved for treatments that demonstrate substantial potential in early clinical trials and is intended to expedite drug development and review [31]. Lanifibranor is a pan-PPAR (peroxisome proliferator-activated receptor) agonist that modulates key metabolic, inflammatory, and fibrogenic pathways in the pathogenesis of MASH [31]. Initial findings in the phase 2b, double-blind, placebo controlled NATIVE trial showed that lanifibranor (1200 mg and 800 mg) had a significant improvement in steatosis, activity, fibrosis (SAF) scores when compared to placebo. The SAF score (0–4) is a histologic scoring system where higher values reflect more active disease [32].

The NATIVE trial enrolled a total of 247 patients who underwent randomization; 103 had type 2 diabetes mellitus and 188 (76%) had significant (moderate) or advanced fibrosis. Compared with the placebo group, the percentage of patients who had a decrease of at least 2 points in the SAF score without worsening fibrosis was much higher in the 1200 mg lanifibranor group than those who took 800 mg of lanifibranor [32]. Although the drug’s effects were modest with the lower dose, the results still favored the treatment group over the placebo. In addition, the lanifibranor group had decreased liver enzyme levels and improvement in the majority of lipid, inflammatory, and fibrosis markers [32].

These encouraging findings paved the way for advancement to a Phase 3 trial (NATiV3), which recently completed enrollment [33]. Final results are anticipated in the first half of 2026 and will be pivotal in determining whether lanifibranor gains regulatory approval. Out of the current treatment landscape, lanifibranor shows huge promise as an emerging therapy specifically for MASH [33]. If approved, it could significantly expand the therapeutic arsenal available for patients with MASLD.

### 4.3. Fibroblast Growth Factor (FGF) Analogs

Fibroblast growth factor (FGF)-based agents represent an emerging class of therapeutics showing promise in the treatment of MASLD and MASH. Fibroblast growth factors and their receptors play an important role in maintaining metabolic homeostasis in the liver. Dysregulation in these pathways have been implicated in contributing to hepatic lipid accumulation and chronic inflammation leading to MASLD and MASH, respectively [34]. In the liver, fibroblast growth factors (FGF19, FGF21, FGF23), have been shown to regulate hepatic lipid metabolism, fasting response and bile acid homeostasis [34]. Therapeutics focusing on FGF21 have been of particular interest as up-regulation in FGF21 has been linked to progression of fatty liver disease. In obese children, elevated expression of FGF21 has been confirmed as a risk factor for steatosis [34].

As a result, pegbelfermin, which is a recombinant PEGylated analog of human FGF21 showed great initial results when treating patients with MASH and stage 2 fibrosis. The medication exerted its effects by increasing adiponectin and decrease in serum pro-C3, which yielded in decreased liver fat, transaminases and liver stiffness as assessed by MR elastography [34]. Despite showing early promise, the results of the phase 2b FALCON trials did not yield favorable outcomes when comparing to placebo and as a result, further trials of the drug were abated [35].

Efruxifermin, another FGF21 analog, has shown promising results in recent phase 2 trials. Although initial studies did not demonstrate significant improvement in fibrosis scores compared to placebo [36], efruxifermin showed favorable trends in steatosis reduction, liver enzyme normalization, and non-invasive fibrosis markers. Consequently, it has progressed to a robust phase 3 clinical development program (SYNCHRONY). This includes trials focused on both fibrosis regression in F2–F3 patients and treatment of compensated cirrhosis (F4) [37]. The SYNCHRONY program is actively enrolling patients, with results anticipated by 2032 [38].

In addition to FGF21-based agents, aldafermin (NGM282), an analog of FGF19, has demonstrated therapeutic potential for MASLD. This medication exerts its effects on FGF19 which suppresses bile acid synthesis via CYP7A1, thereby reducing bile acid-mediated hepatotoxicity. It also improves metabolic parameters, including insulin sensitivity and liver fat content [39]. In a recent retrospective trial, 491 patients were included where Aldafermin showed a dose-dependent reduction in liver fat content, alanine aminotransferase levels (ALT), aspartate aminotransferase levels (AST) and enhanced liver fibrosis scores, in the 1 mg and 3 mg subgroups [39]. However, the treatments impact on improving histologic fibrosis lacked statistical significance. As a result, larger and longer trials are needed to effectively establish the robustness of this drug therapy.

## 5. Discussion

As the incidence of MASLD rises worldwide, patients are at an increased risk of developing sequalae of progressive liver disease including liver cirrhosis, hepatocellular carcinoma (HCC), and liver related mortality. The key to prevention will be to reduce the risk of developing metabolic syndrome, which culminates from a cluster of conditions including type 2 DM, hyperlipidemia, central obesity, and hypertension. These risk factors are well-established contributors to the development and progression of MASLD, making metabolic control a vital component of any therapeutic approach.

As various therapies still remain under investigation, there have been promising drug developments that will make treating MASLD less challenging. Resmetirom, GLP-1 receptor agonists, lanifibranor, and efruxifermin are some of the few that have shown favorable clinical trial results. Notably, Resmetirom has emerged as the therapy of choice as it is the first and only FDA approved medication available to treat MASLD. However, given the complex and multifactorial pathophysiology of MASLD, it is unlikely that a single therapy will serve as a universal solution.

Looking ahead, multi-pathway approaches such as combination regimens pairing antifibrotic agents with metabolic modulators may prove more effective than single-drug strategies, especially for patients with advanced fibrosis or significant cardiometabolic risk. Advances in non-invasive biomarkers and imaging modalities will also be crucial, enabling earlier detection of therapeutic response and reducing reliance on serial liver biopsies.

Thus, it will be important to closely follow phase 3 trial results of multiple medications that remain in the pipeline. For years, the treatment options for MASLD were limited and largely supportive. Today, as targeted therapies continue to emerge, we are witnessing a shift toward disease-modifying interventions that not only improve liver histology and fibrosis but also address the broader cardiometabolic risks that accompany MASLD. This evolving treatment landscape offers new hope for patients and clinicians alike in the fight against the global liver disease epidemic.

## Figures and Tables

**Table 1:** Comparative effects of anti-diabetic drugs [3,4,5,6,7,8,9,10,11,12,13,14,15,16,17,18,19,20,21,22,23,24,25,26,27].

Drug Class	Liver Fibrosis	Hepatic Steatosis	Body Weight	Cardiorenal Effects	MASH Resolution
**GLP-1 Receptor Agonists** (e.g., Semaglutide, Tirzepatide, Cotadutide)	Strong improvement in fibrosis markers and histology	Strong reduction in liver fat content	Consistent and clinically meaningful weight loss	Significant cardiovascular and potential renal benefits	High rates of steatohepatitis resolution in trials
**SGLT-2 Inhibitors** (e.g., Empagliflozin, Dapagliflozin)	Modest improvement (evidence low-to-moderate certainty)	Mild reduction in steatosis	Moderate weight loss	Strong and well-established cardiorenal protection	Limited evidence for MASH resolution
**DPP-IV Inhibitors** (e.g., Sitagliptin, Saxagliptin)	No significant effect	No meaningful impact	Weight-neutral	Generally safe with modest metabolic benefit	No evidence for MASH resolution
**Pioglitazone**	Moderate improvement in fibrosis	Moderate reduction in steatosis	Often associated with weight gain	Some cardiorenal benefit but risk of heart failure	Moderate rates of MASH resolution
**Metformin**	No proven direct effect	Mild indirect improvement via insulin sensitization	Weight-neutral to mild loss	No established cardiorenal benefit	No clear evidence for MASH resolution
**Lanifibranor**	Moderate to strong improvement in fibrosis (dose-dependent)	Moderate to strong reduction in steatosis and SAF score	Mild weight loss or neutral effect	Favorable lipid and inflammation profile	Moderate to strong resolution rates without worsening fibrosis in higher-dose arm
**FGF Analogs** (e.g., Efruxifermin, Aldafermin)	Moderate improvement in fibrosis (stronger in some FGF19 analog studies)	Moderate to strong liver fat reduction on imaging	Mild weight loss or neutral	Potential metabolic benefits; limited direct CV outcome data	Moderate resolution rates, variable by agent

## Data Availability

Not applicable.

## References

[1] Rinella M.E., Lazarus J.V., Ratziu V., Francque S.M., Sanyal A.J., Kanwal F., Romero D., Abdelmalek M.F., Anstee Q.M., Arab J.P. (2023). A multisociety Delphi consensus statement on new fatty liver disease nomenclature. Hepatology.

[2] Le P., Tatar M., Dasarathy S., Alkhouri N., Herman W.H., Taksler G.B., Deshpande A., Ye W., Adekunle O.A., McCullough A. (2025). Estimated Burden of Metabolic Dysfunction–Associated Steatotic Liver Disease in US Adults, 2020 to 2050. JAMA Netw. Open..

[3] Abdelhameed F., Mustafa A., Kite C., Lagojda L., Dallaway A., Than N.N., Kassi E., Kyrou I., Randeva H.S. (2025). Gut Microbiota and Metabolic Dysfunction-Associated Steatotic Liver Disease (MASLD): Emerging Pathogenic Mechanisms and Therapeutic Implications. Livers.

[4] Younossi Z.M., Koenig A.B., Abdelatif D., Fazel Y., Henry L., Wymer M. (2023). Changing epidemiology, global trends and implications for outcomes of NAFLD. J. Hepatol..

[5] Kudaravalli P., John S. (2025). Nonalcoholic Fatty Liver. StatPearls [Internet].

[6] Rinella M.E., Neuschwander-Tetri B.A., Siddiqui M.S., Abdelmalek M.F., Caldwell S., Barb D., Kleiner D.E., Loomba R. (2023). AASLD Practice Guidance on the clinical assessment and management of nonalcoholic fatty liver disease. Hepatology.

[7] Petagine L., Zariwala M.G., Patel V.B. (2023). Non-alcoholic fatty liver disease: Immunological mechanisms and current treatments. World J. Gastroenterol..

[8] Chalasani N., Younossi Z., Lavine J.E., Diehl A.M., Brunt E.M., Cusi K., Charlton M., Sanyal A.J. (2012). The diagnosis and management of non-alcoholic fatty liver disease: Practice Guideline by the American Association for the Study of Liver Diseases, American College of Gastroenterology, and the American Gastroenterological Association. Hepatology.

[9] Musso G., Cassader M., Rosina F., Gambino R. (2012). Impact of current treatments on liver disease, glucose metabolism and cardiovascular risk in non-alcoholic fatty liver disease (NAFLD): A systematic review and meta-analysis of randomised trials. Diabetologia.

[10] Stine J.G., Long M.T., Corey K.E., Sallis R.E., Allen A.M., Armstrong M.J., Conroy D.E., Cuthbertson D.J., Duarte-Rojo A., Hallsworth K. (2023). American College of Sports Medicine (ACSM) International Multidisciplinary Roundtable report on physical activity and nonalcoholic fatty liver disease. Hepatol. Commun..

[11] Verrastro O., Panunzi S., Gissey-Castragneto L., de Gaetano A., Lembo E., Capristo E., Guidone C., Angelini G., Pennestrì F., Sessa L. (2023). Bariatric-metabolic surgery vs. lifestyle intervention plus best medical care in non-alcoholic steatohepatitis (BRAVES): A multi-centre, open-label, randomized trial. Lancet.

[12] Krishnan A., Hadi Y., Alqahtani S.A., Woreta T.A., Fang W., Abunnaja S., Szoka N., Tabone L.E., Thakkar S., Singh S. (2023). Cardiovascular Outcomes and Mortality After Bariatric Surgery in Patients With Nonalcoholic Fatty Liver Disease and Obesity. JAMA Netw. Open..

[13] Koullias E., Papavdi M., Koskinas J., Deutsch M., Thanopoulou A. (2025). Targeting Metabolic Dysfunction-Associated Steatotic Liver Disease (MASLD): Available and Future Pharmaceutical Options. Cureus.

[14] Boer G.A., Hay D.L., Tups A. (2023). Obesity pharmacotherapy: Incretin action in the central nervous system. Trends Pharmacol. Sci..

[15] Sanyal A.J., Newsome P.N., Kliers I., Østergaard L.H., Long M.T., Kjær M.S., Cali A.M., Bugianesi E., Rinella M.E., Roden M. (2025). Phase 3 Trial of Semaglutide in Metabolic Dysfunction-Associated Steatohepatitis. N. Engl. J. Med..

[16] Aronne L.J., Horn D.B., le Roux C.W., Ho W., Falcon B.L., Valderas E.G., Das S., Lee C.J., Glass L.C., Senyucel C. (2025). SURMOUNT-5 Trial Investigators. Tirzepatide as Compared with Semaglutide for the Treatment of Obesity. N. Engl. J. Med..

[17] Nahra R., Wang T., Gadde K.M., Oscarsson J., Stumvoll M., Jermutus L., Hirshberg B., Ambery P. (2021). Effects of Cotadutide on Metabolic and Hepatic Parameters in Adults With Overweight or Obesity and Type 2 Diabetes: A 54-Week Randomized Phase 2b Study. Diabetes Care..

[18] Boehringer Ingelheim Boehringer Receives U.S. FDA Breakthrough Therapy Designation and Initiates Two Phase III Trials in MASH for Survodutide. https://www.boehringer-ingelheim.com/human-health/metabolic-diseases/survodutide-us-fda-breakthrough-therapy-phase-3-trials-mash.

[19] Sanyal A.J., Bedossa P., Fraessdorf M., Neff G.W., Lawitz E., Bugianesi E., Anstee Q.M., Hussain S.A., Newsome P.N., Ratziu V. (2024). 1404-0043 Trial Investigators. A Phase 2 Randomized Trial of Survodutide in MASH and Fibrosis. N. Engl. J. Med..

[20] Singh A., Sohal A., Batta A. (2024). GLP-1, GIP/GLP-1, and GCGR/GLP-1 receptor agonists: Novel therapeutic agents for metabolic dysfunction-associated steatohepatitis. World J. Gastroenterol..

[21] Aldhaleei W.A., Abegaz T.M., Bhagavathula A.S. (2024). Glucagon-like Peptide-1 Receptor Agonists Associated Gastrointestinal Adverse Events: A Cross-Sectional Analysis of the National Institutes of Health All of Us Cohort. Pharmaceuticals.

[22] Chan W.-K., Chuah K.-H., Rajaram R.B., Lim L.-L., Ratnasingam J., Vethakkan S.R. (2023). Metabolic Dysfunction-Associated Steatotic Liver Disease (MASLD): A State-of-the-Art Review. J. Obes. Metab. Syndr..

[23] Chopra S., Lai M. Management of Metabolic Dysfunction Associated Steatotic Liver Disease (Nonalcoholic Fatty Liver Disease) in Adults. *UpToDate*. Updated 9 February 2025. https://www.uptodate.com/contents/management-of-metabolic-dysfunction-associated-steatotic-liver-disease-nonalcoholic-fatty-liver-disease-in-adults?.

[24] Belfort R., Harrison S.A., Brown K., Darland C., Finch J., Hardies J., Balas B., Gastaldelli A., Tio F., Pulcini J. (2006). A placebo-controlled trial of pioglitazone in subjects with nonalcoholic steatohepatitis. N. Engl. J. Med..

[25] Michalopoulou E., Thymis J., Lampsas S., Pavlidis G., Katogiannis K., Vlachomitros D., Katsanaki E., Kostelli G., Pililis S., Pliouta L. (2025). The Triad of Risk: Linking MASLD, Cardiovascular Disease and Type 2 Diabetes; From Pathophysiology to Treatment. J. Clin. Med..

[26] Ong Lopez A.M.C., Pajimna J.A.T. (2024). Efficacy of sodium glucose cotransporter 2 inhibitors on hepatic fibrosis and steatosis in non-alcoholic fatty liver disease: An updated systematic review and meta-analysis. Sci. Rep..

[27] Perumpail B.J., Li A.A., John N., Sallam S., Shah N.D., Kwong W., Cholankeril G., Kim D., Ahmed A. (2018). The Role of Vitamin E in the Treatment of NAFLD. Diseases.

[28] Song Y., Ni W., Zheng M., Sheng H., Wang J., Xie S., Yang Y., Chi X., Chen J., He F. (2025). Chinese NAFLD Clinical Research Network (CNAFLD CRN). Vitamin E (300 mg) in the treatment of MASH: A multi-center, randomized, double-blind, placebo-controlled study. Cell Rep. Med..

[29] Abera M., Suresh S.B., Malireddi A., Boddeti S., Noor K., Ansar M., Malasevskaia I. (2024). Vitamin E and Non-alcoholic Fatty Liver Disease: Investigating the Evidence Through a Systematic Review. Cureus.

[30] Harrison S.A., Taub R., Neff G.W., Lucas K.J., Labriola D., Moussa S.E., Alkhouri N., Bashir M.R. (2023). Resmetirom for nonalcoholic fatty liver disease: A randomized, double-blind, placebo-controlled phase 3 trial. Nat. Med..

[31] Inventiva Inventiva Receives FDA Breakthrough Therapy Designation for Lead Drug Candidate Lanifibranor in NASH. Sofinnova Partners. 12 October 2020. https://sofinnovapartners.com/news/inventiva-receives-fda-breakthrough-therapy-designation-for-lead-drug-candidate-lanifibranor-in-nash?utm_source=chatgpt.com.

[32] Francque S.M., Bedossa P., Ratziu V., Anstee Q.M., Bugianesi E., Sanyal A.J., Loomba R., Harrison S.A., Balabanska R., Mateva L. (2021). A randomized, controlled trial of the pan-PPAR agonist lanifibranor in NASH. N. Engl. J. Med..

[33] Inventiva *Inventiva Announces Completion of Enrollment in the Phase 3 NATiV3 Clinical Trial of Lanifibranor in Patients with MASH and Advanced Fibrosis*; BioSpace (Daix, France): 2025. https://www.biospace.com/press-releases/inventiva-announces-completion-of-enrollment-in-the-phase-3-nativ3-clinical-trial-of-lanifibranor-in-patients-with-mash-and-advanced-fibrosis.

[34] Ocker M. (2020). Fibroblast growth factor signaling in non-alcoholic fatty liver disease and non-alcoholic steatohepatitis: Paving the way to hepatocellular carcinoma. World J. Gastroenterol..

[35] Abdelmalek M.F., Sanyal A.J., Nakajima A., Neuschwander-Tetri B.A., Goodman Z.D., Lawitz E.J., Harrison S.A., Jacobson I.M., Imajo K., Gunn N. (2024). Pegbelfermin in Patients With Nonalcoholic Steatohepatitis and Compensated Cirrhosis (FALCON 2): A Randomized Phase 2b Study. Clin. Gastroenterol. Hepatol..

[36] Noureddin M., Rinella M.E., Chalasani N.P., Neff G.W., Lucas K.J., Rodriguez M.E., Rudraraju M., Patil R., Behling C., Burch M. (2025). Efruxifermin in Compensated Liver Cirrhosis Caused by MASH. N. Engl. J. Med..

[37] Akero Therapeutics (2025). Clinical Trials—Efruxifermin for MASH. Akero Therapeutics Website. https://akerotx.com/clinical-trials/.

[38] Akero Therapeutics (2025). A Phase 3, Randomized, Double-Blind, Placebo-Controlled Study Evaluating the Safety and Efficacy of Efruxifermin in Subjects with Non-Cirrhotic NASH/MASH and fibrosis (ClinicalTrials.gov Identifier: NCT06215716). NCT06215716.

[39] Marey M.M., Belal M., Awad A.A., Rabea E.M., Hassan M.A., Abbas A.W., Nashwan A.J. (2024). Efficacy and safety of aldafermin in non-alcoholic steatohepatitis: A systematic review and meta-analysis of randomized controlled trials. Clin. Res. Hepatol. Gastroenterol..

